# Gas-Sensitive Characteristics of Graphene Composite Tungsten Disulfide to Ammonia

**DOI:** 10.3390/s22228672

**Published:** 2022-11-10

**Authors:** Fei Zhao, Zhongxue Li, Yongzhong Fu, Quan Wang

**Affiliations:** 1 Institute of Electrical and Information Engineering, Zhenjiang College, Zhenjiang 212100, China; 2 Zhenjiang Key Laboratory of Advanced Sensing Materials and Devices, School of Mechanical Engineering, Jiangsu University, Zhenjiang 212013, China; 3 State Key Laboratory of Transducer Technology, Chinese Academy of Sciences, Shanghai 200050, China

**Keywords:** graphene, WS_2_, first principles, gas sensor

## Abstract

Two-dimensional materials have outstanding application prospects in gas sensing. By constructing composite structures of various gas-sensitive materials, more-efficient and sensitive gas sensors can be further developed. After graphene is compounded with WS_2_, the composite material can improve the gas detection performance. In this work, the adsorption energy and the electronic properties of a graphene/WS_2_ structure were calculated by first-principles before and after adsorption of NH_3_. The calculation results indicate that the bandgap of the material was appreciably reduced after NH_3_ was adsorbed. In addition, a graphene/WS_2_ gas sensor was prepared. The response of the sensor to NH_3_ at a concentration of 100 ppm was 2.42% and 1.73% at 30 °C and 60 °C, respectively. Combining simulation with experiment, it is feasible to use graphene composite WS_2_ to detect NH_3_, which provides a new idea for applications of graphene and other composite materials in gas sensing.

## 1. Introduction

In recent years, in-depth research on two-dimensional materials has promoted further development of gas sensor technology, and has enabled gas sensors to move toward high sensitivity, stability, and reliability, as well as a fast response and recovery rate [1,2,3]. Among two-dimensional materials, graphene has the advantages of exhibiting a unique single-atomic-layer structure and a specific surface area that is close to the theoretical value, which is well-suited for applications in gas detection [4].

However, because of limitations imparted by the electrical properties of graphene, the performance of gas sensors prepared by using graphene has thus far failed to meet theoretical expectations. Accordingly, researchers have prepared graphene-based composite materials (such as doping atoms (e.g., B, N, and Pt), metal oxides, and functional polymers on the surface of graphene). Rapid transfer of charges at the interface of the composite material can improve the sensing performance [5,6,7]. In addition, two-dimensional transition metal chalcogenides (TDMs) also have outstanding application prospects in gas sensing. For example, sensors constructed with MoS_2_, MoTe_2_, and other materials have advantages of, e.g., high sensitivity and function at room temperature [8,9].

As a representative material in TDMs, during electrical testing of WS_2_, a dense layered stacked structure readily forms in the process of forming a charged network inside the material. This stacked structure is not conducive to the contact between the nanosheets and gas molecules [10]. Alternatively, researchers can combine WS_2_ with graphene to prepare a graphene/WS_2_ composite structure. The large specific surface area of graphene can absorb more gas molecules, whereas WS_2_ can output a higher response. Complementing the advantages of the two materials will facilitate gas detection [11,12].

This work discusses adsorption of NH_3_ based on a graphene/WS_2_ composite structure. The electronic energy band structures of WS_2_, graphene/WS_2_, and graphene/WS_2_/NH_3_ were calculated by using first-principles, and the bandgap changes were compared and analyzed. Moreover, a graphene/WS_2_ composite gas sensor was prepared, and the response sensitivity of the device to NH_3_ at various temperatures was discussed. A combination of theoretical simulation and experiment helps to better understand detection of NH_3_ by the graphene/WS_2_ composite structure.

## 2. Materials and Methods

### 2.1. Simulation Model and Calculation Method

**Figure 1:**
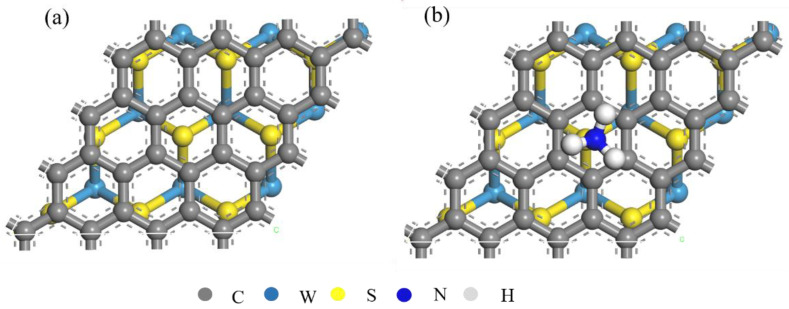
Top view of graphene/WS_2_ composite structure matching mode. (**a**) Before adsorbing NH_3_, and (**b**) after adsorbing NH_3_.

The research goal is adsorption of ammonia gas by using a graphene/WS_2_ composite structure; its properties were studied by using CASTEP based on density functional theory [13,14]. To ensure the accuracy and reliability of the calculation results, a generalized gradient approximation combined with a Perdew–Burke–Ernzerh function was used to describe electronic exchange and correlation. The Tkatchenko–Scheffler method was used for dispersion correction [15]. The Brillouin zone of the system used a 5 × 5 × 1 k-point grid, and the self-consistent convergence accuracy and force field convergence accuracy was set to 5 × 10^−5^ and 1 × 10^−1^ eV/nm, respectively. The corresponding maximum stress displacement did not exceed 0.02 GPa and 5 × 10^−4^ nm, respectively. The BFGS algorithm was used for geometric optimization [16]. Figure 1 shows the simulation model. The composite structure supercell model was composed of 3 × 3 × 1 single-layer tungsten disulfide and 4 × 4 × 1 graphene supercells. The thickness of the vacuum layer was 2 nm, and the optimized layer spacing was 0.34 nm. Generally, the hydrogen atom in NH3 and the sulfur atom in WS2 show high binding energy. The adsorption position is determined by the binding energy of chemical bonds between NH3 and WS2.

### 2.2. Experimental Steps

The graphene grown by CVD was transferred onto an Si/SiO_2_ substrate by using wet transfer technology. A comb-shaped electrode was deposited onto the substrate. The space of the electrode was 200 μm. WS_2_ nanosheets were prepared by a one-step hydrothermal reaction. Tungsten chloride (WCl6, 2.5 mmol) and 25 mmol of thioacetamide (CH_3_CSNH_2_) were dissolved in 35 mL of deionized water and stirred for 40 min; then, the solution was transferred to a Teflon-lined stainless steel autoclave, held at a temperature of up to 220 °C, and kept at this temperature for 24 h. After cooling, the reaction products were washed and freeze-dried. Then, WS_2_ was ultrasonically dispersed in deionized water. The concentration of the WS_2_ suspension was 10 mg/mL. A 1.5-µL pipette was used to apply one to two drops of WS_2_ suspension onto the graphene above the substrate. After drying at room temperature in air, it was aged at 60 °C for 2 h to achieve stability. After the device was prepared, a WS-30B system was used to test the response characteristics of the graphene/WS_2_ composite gas sensor in a 100 ppm NH_3_ atmosphere. The temperature, humidity and gas concentration are automatically controlled by the gas sensing test bench, and the atmosphere of the test chamber is controlled by software. To reduce the influence of humidity in the experiment, the humidity of the test system was controlled to 25%, and the test temperatures were 30 °C and 60 °C. Before the test, background gas (e.g., dry air) was introduced, and then NH_3_ was introduced for testing and signal acquisition.

## 3. Results and Discussion

### 3.1. Discussion of Band Structure

**Figure 2:**
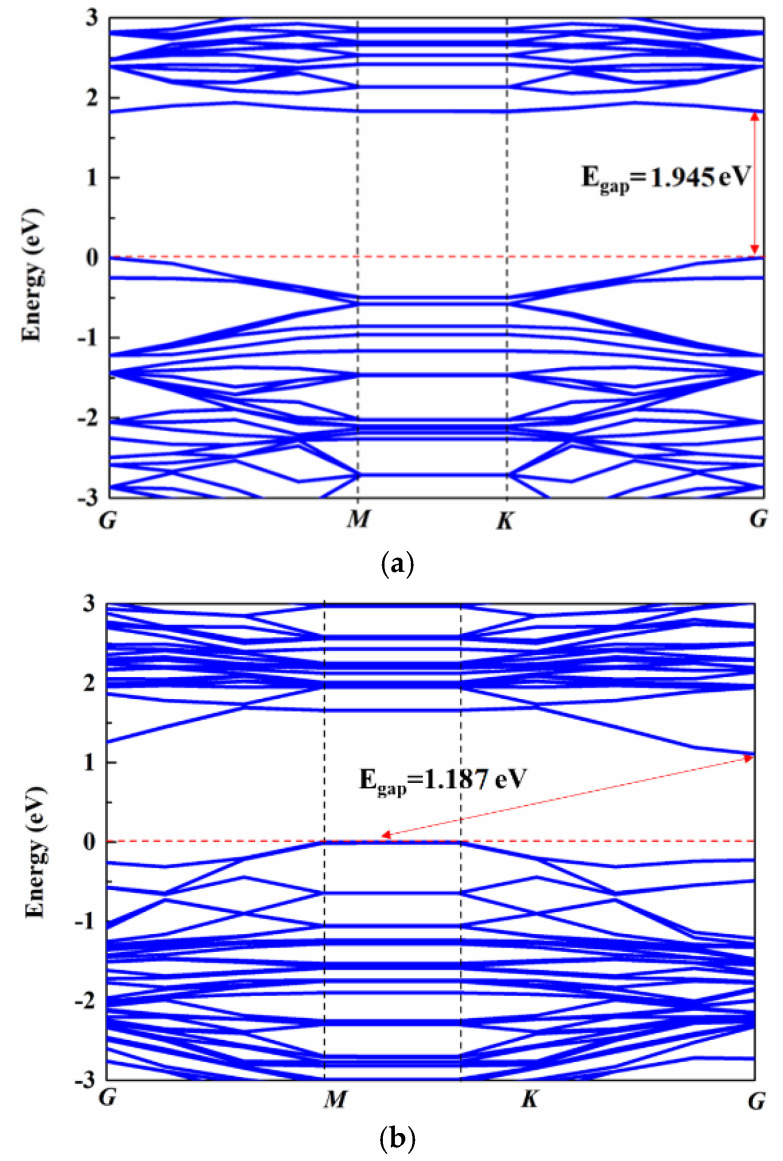

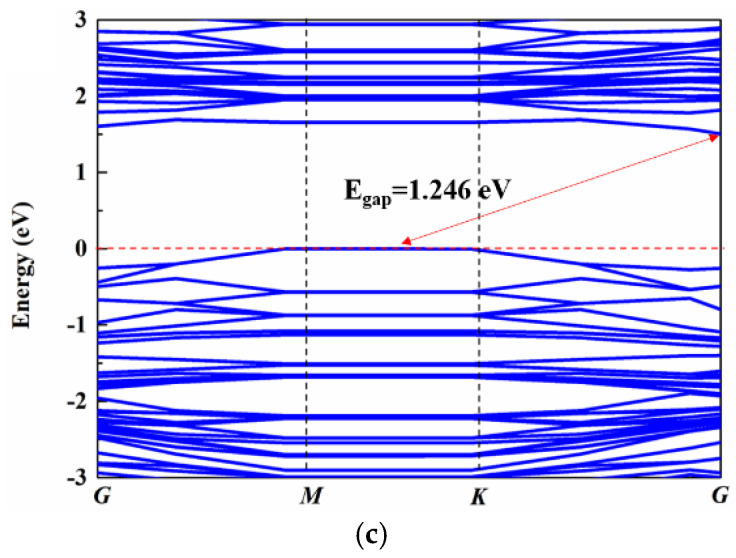
Energy band structure of WS_2_ and graphene/WS_2_ composites. (**a**) Energy band of WS_2_, energy band of graphene/WS_2_ composites (**b**) before adsorbing NH_3_, and (**c**) after adsorbing NH_3_.

The interfacial interactions of graphene/WS_2_ directly affect the electron transport properties inside the material. Therefore, the energy band structure of the composite material before and after the adsorption of gas was calculated. The bandgap of graphene is zero, and thus in this work only the energy band of WS_2_ and graphene/WS_2_ was calculated. Figure 2a maps the energy band structure of WS_2_. Figure 2a indicates that the bandgap of WS_2_ was 1.945 eV. Both the bottom of the conduction band and the top of the valence band are located at point G, indicating that WS_2_ is a direct bandgap. This value is similar to previous results [17,18], which also indicates that the parameters of the simulation settings conform to the simulation requirements.

Figure 2b,c show the band structure of graphene composite WS_2_ before and after adsorption of NH_3_. When graphene was compounded with WS_2_, the bandgap of the composite structure changed significantly. The top of the valence band was at the high symmetry point G, and the bottom of the conduction band was at point G. The composite structure transformed into an indirect bandgap; the bandgap value was 1.187 eV. Concomitantly, the independent energy band structure of WS_2_ was largely retained when the two materials were composited. The conduction band shifted to the Fermi level with a low bias, such as in n-type doping. This is attributable to the electrons provided by the graphene transition to the conduction band. The energy height of the electrons that left the bottom of the conduction band was reduced. When the composite structure adsorbed NH_3_ molecules, its energy band structure did not change significantly compared with the structure of not-yet-adsorbed gas; but the bandgap value decreased to 1.246 eV, and the conduction band continued to move toward the Fermi level. Thus, after NH_3_ was adsorbed, electrons more readily migrated from the top of the valence band to the low conduction band, and the conductivity of the composite material was significantly improved [19].

**Figure 3:**
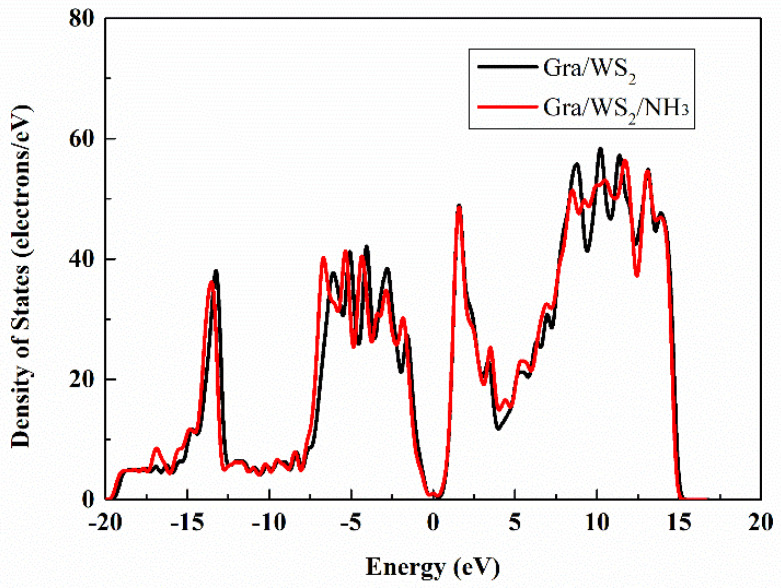
Calculation of the total density of states of graphene/WS_2_ before and after NH_3_ adsorption.

Figure 3 shows calculations of the total density of states of graphene/WS_2_ before and after adsorption of NH_3_. After adsorption of NH_3_, the total density of states shifted slightly to the left, which indicates that charge transfer was facilitated [20]. Concomitantly, a small peak was evident near −17 eV. The density of states decreased at a peak near 8–11 eV to the right of the Fermi level. Introduction of NH_3_ changed the strong hybridization and bonding between the atomic orbitals, which changed the charge transfer. In addition, under this simulation system, the adsorption energy E_ad_ of graphene/WS_2_ for NH_3_ can be calculated as follows [21,22].

E_ad_ = E(WS_2_/graphene/NH_3_) − E(WS_2_/graphene) − E(NH_3_) (1)

In this simulation system, the total energy $E\left({\mathrm{WS}}_{2}/{\mathrm{graphene}/\mathrm{NH}}_{3}\right)$ of the material after NH_3_ adsorption was −94,137.75 eV, whereas the total energy of the composite material without adsorbed gas $E\left({\mathrm{WS}}_{2}/\mathrm{graphene}\right)$ and $E\left({\mathrm{NH}}_{3}\right)$ was −93,812.52 and −323.98 eV, respectively. The adsorption energy E_ad_ of graphene/WS_2_ to NH_3_ was calculated as −1.25 eV. This value indicates that the composite material can readily achieve NH_3_ adsorption.

Through analysis of the simulation results, it was evident that after graphene was compounded with WS_2_, the electron transport inside the material changed. Moreover, the composite material readily adsorbed NH_3_ and the bandgap of the adsorbed material was reduced. This indicates that the composite of graphene and WS_2_ will facilitate construction of a sensor for detecting NH_3_.

### 3.2. Gas Sensitivity Test Results

**Figure 4:**
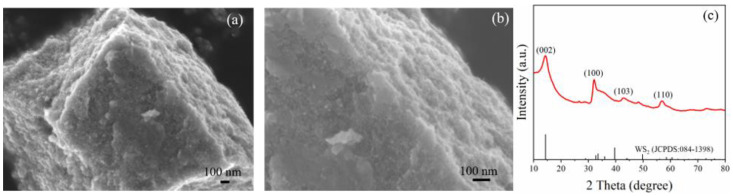
WS_2_ (**a**,**b**) SEM images. (**c**) XRD.

Figure 4a,b show SEM images of pure WS_2_ prepared by the hydrothermal method. Figure 4a indicates that the surface of WS_2_ was composed of agglomerated flocculent particles. After partial magnification in Figure 4b, many WS_2_ nanosheets were found at the edge of the WS_2_ block and presented a loose structure, indicating the high specific surface area, which was beneficial to NH_3_ adsorption and improving the gas sensitivity. Figure 4c shows the XRD profile of as-prepared WS_2_, and the XRD pattern is consistent with pure hexagonal WS_2_ (JPCDS No. 084-1398). The reflections at 14.22°, 34.04°, and 56.78° (corresponding to the (002), (100), and (110) planes, respectively) confirm the presence of the hexagonal phase. The sharp peaks indicate the crystallinity of the as-prepared sample. Some peaks were evident, along with slight rightward shifts of the peak positions and broadening, which indicates that there was some residual compressive stress on the surface of the WS_2_ nanosheets after freeze-drying.

**Figure 5:**
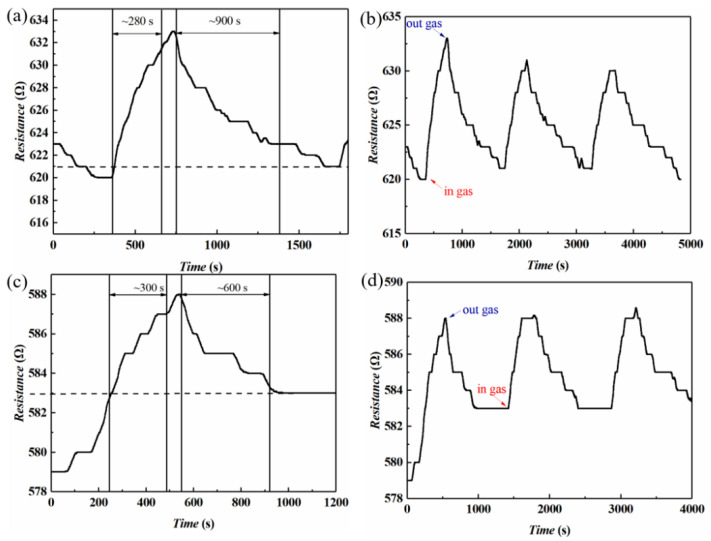
Gas sensitivity of graphene/WS_2_ composites to 100-ppm NH_3_. (**a**) Response/recovery curve and (**b**) repeatability at 30 °C. (**c**) Response/recovery curve and (**d**) repeatability at 60 °C.

Figure 5 shows the single-gas sensitivity response/recovery curve of the graphene/WS_2_ gas sensor when exposed to 100-ppm NH_3_ at 30 °C with 25% humidity. In accordance with the NH_3_ introduction time, the resistance of the gas sensor first increased and then decreased. This trend indicates that the device adsorbed gas molecules in an NH_3_ atmosphere, and detected NH_3_. As the concentration of gas absorbed onto the surface of the graphene and WS_2_ increased, charge transfer occurred between the gas molecules and the composite material. The type and concentration of carriers inside the composite material changed, which eventually led to the change in sensor resistance. This change corresponds to the gas response of the device [23].

The response time (recovery time) was defined as the time required to reach 90% of the change in sensor resistance upon exposure to the target gas (air). Figure 5a indicates that the response time of the sensor was ca. 280 s. After terminating the gas supply, the gas concentration absorbed by the sensor decreased. The gas molecules began to desorb from the surface of the material, and the resistance value of the sensor gradually recovered. This process is gas recovery. The recovery time of this device was ca. 900 s.

A single response/recovery curve can indicate the response and recovery time of the device, but the overall performance of the device must be measured over a long period of time. During the entire experiment, the humidity was set to be 25% and the temperature was 30 °C, which eliminated the interference that would have been caused by temperature and humidity changes. Figure 5b shows multiple response/recovery tests of the gas sensor and the test duration exceeded 1 h. The manifestation of three peaks in Figure 5b indicates that the device experienced three responses/recoveries. In addition, the overall change of the curve was nearly similar, indicating that the sensor had a degree of stability. However, because some gas molecules cannot be completely desorbed, there will be residual gas molecules on the surface of the material. Therefore, after recovery, the resistance value of the device cannot reach the initial value. During the test, the sensor first adsorbed the gas and then desorbed it. After the gas was adsorbed, the resistance of the device increased. However, the resistance value decreased after the gas was introduced. This may be caused by slight fluctuations in the experimental environment during the preliminary test. With the extension of the test time, the sensor can still maintain a good response. However, the peak value of the resistance exhibited a downward trend. There are many impurities in the wet-transferred graphene. The heat caused by the current can eliminate impurities on the surface of the material and improve the contact. Consequently, the resistance value can be reduced. In addition, part of the gas adsorbed onto the WS_2_ sheet failed to desorb, which reduced the resistance of the WS_2_, improved the barrier between graphene and WS_2_, accelerated the transfer between electrons, and changed the overall electrical performance of the devices [24]. 

Temperature corresponds to the diffusion rate of gas molecules. The detection performance of the sensor differed at different temperatures. Therefore, after adjusting the temperature to 60 °C, one can test the sensor again. Other experimental conditions are the same as those at 30 °C.

Figure 5c shows a single-gas sensitivity response/recovery curve of the graphene/WS_2_ gas sensor when the NH_3_ concentration was 100 ppm and temperature was 60 °C. Similarly to the test result at 30 °C, the sensor still detected NH_3_ molecules at 60 °C. At 60 °C, the response time of the gas sensor was 300 s and the recovery time was 600 s. The response time was the same as that at 30 °C, but the recovery time was significantly shortened. This is because the increase in temperature aggravated the movement of NH_3_ molecules. Molecules that might be attached to the gas-sensitive material at 30 °C will also accelerate desorption as the temperature increases. In accordance with the various means by which gas molecules are adsorbed on the surface of the material, the means of gas adsorption can be divided into physical adsorption and chemical adsorption. Physical adsorption mainly depends on the interaction forces between molecules, whereas chemical adsorption depends on the formation of chemical covalent bonds between gas molecules and the surface of the composite material. In general, physical adsorption precedes chemical adsorption [25]. The temperature increased by 30 °C, and the desorption time was reduced by 30%. This result indicates that desorption was unstable, and that changes in the environment can affect the performance of the device. The aforementioned result also indirectly indicates that many stable chemical bonds have not been formed between the gas molecules and materials. NH_3_ molecules mostly remain on the surface of composite materials by physical adsorption.

Figure 5d shows a gas sensitivity response/recovery curve of the gas sensor when the NH_3_ concentration was 100 ppm and the temperature was 60 °C. The device responded and recovered many times within more than 1 h of test time.

Figure 5d indicates that the trend of the curve was relatively stable, indicating that the device can work stably at 60 °C. Distinct from the change after the test at 30 °C, the resistance value of the sensor remained stable for some time after returning to the lowest value. The reason for this result is that higher temperature accelerated adsorption of NH_3_ such that NH_3_ underwent partial chemical adsorption concomitantly with physical adsorption. Over the course of desorption, a temperature of 60 °C is insufficient to destroy the stable chemical covalent bond. Chemical adsorption of NH_3_ on the surface of the material prevented the device from responding in the next cycle. As a result, there was a smooth region below the curve. 

The device can maintain the excellent response after passing many cycle tests. Due to multiple recovery processes, many NH_3_ molecules that were not desorbed gathered on the surface of the composite materials. The aggregated molecules hindered the desorption and slowed the recovery rate. Therefore, the recovery time exhibited a gradually increasing trend [26]. 

The response can intuitively reflect the performance of the device. The response (S) of the sensor was defined as (|R_0_ − R_t_|)/R_0_ × 100%. In the aforementioned formula, R_0_ is the initial resistance value of the gas sensor in air and Rt is the resistance value of the gas sensor after adsorbing NH_3_ molecules. At temperatures of 30 °C and 60 °C, the response of the sensor was 2.42% and 1.73%, respectively. The response of this type of device was lower than that in previous studies, but exhibited a faster response time (Table 1).

The low response mainly corresponds to the method used to prepare the device. Especially after one transfers the graphene to the electrode, the graphene will break, curl, and leave contaminants. In addition, the layered structure of WS_2_ is not obvious and there is stacking, which cannot provide sites for more gas molecules. The electron migration ability is reduced, and the composite material fails to assume the role of a bridge for electron transport.

## 4. Conclusions

This work analyzed adsorption of NH_3_ by graphene/WS_2_ composites through simulation. After adsorption of NH_3_, the bandgap value of the material was significantly reduced, and the adsorption energy was calculated: 1.25 eV. The simulation results indicate that the use of graphene composite WS_2_ to detect NH_3_ is feasible under certain conditions. A graphene/WS_2_ gas sensor was also prepared, which realized detection of NH_3_ and verified the assumptions of the simulation results. At 30 °C and 60 °C, the response to 100-ppm NH_3_ was 2.42% and 1.73%, respectively. This research provides new ideas for applications of graphene and other composite materials in gas sensing.

## Figures and Tables

**Table 1:** Gas sensitivity of various types of two-dimensional materials.

Material	Target Gas	Concentration (ppm)	Response (%)	Response Time (s)	Operating Temperature (°C)	
WS_2_ NFs	NH_3_	5	252.4	921	25	[27]
WS_2_/CNFs	NO_2_	50	279.1	54	25	[28]
WS_2_/PbS	NO_2_	5	592.8	612	25	[29]
MoS_2_/GO	NH_3_	100	3.6	2751	150	[30]
SnO_2_/rGO	CH_4_	10000	91.3	426	25	[31]
WS_2_/GO	NH_3_	100	2.4	280	30	This work

## Data Availability

The data that support the findings of this study are available upon reasonable request from the authors.
